# Hoarseness as the first symptom in a patient with acute suppurative thyroiditis secondary to a pyriform sinus fistula: a case report

**DOI:** 10.1186/s12887-023-04089-9

**Published:** 2023-05-30

**Authors:** Toshifumi Otsuki, Ryoichi Onuma, Kohei Tonsho, Dai Saito, Hideyuki Sakai, Miki Kamimura, Yohei Watanabe, Noriko Kurihara, Eiichi Ishida, Satoru Kumaki

**Affiliations:** 1 Department of Pediatrics, National Hospital Organization Sendai Medical Center, 2-11-12, Miyagino, Miyagino-ku, Sendai, 983-8520 Japan; 2 Department of Radiology, National Hospital Organization Sendai Medical Center, Sendai, Japan; 3 Department of Otorhinolaryngology, Head & Neck Surgery, National Hospital Organization Sendai Medical Center, Sendai, Japan

**Keywords:** Pyriform sinus fistula, Hoarseness, Acute suppurative thyroiditis

## Abstract

**Background:**

Pyriform sinus fistulas (PSFs) are rare congenital anomalies of the third or fourth brachial pouch. Dyspnea is reportedly secondary to compression by a neck mass. However, hoarseness, as the first symptom of PSF, has not yet been reported.

**Case presentation:**

This report describes an 11-year-old girl presenting with hoarseness as the first symptom of PSF. Hoarseness occurred 2 days prior to admission. On admission, she had fever, hoarseness, and an elastic soft mass on her left anterior neck. Contrast-enhanced computed tomography of the cervical region demonstrated an abscess partially infiltrating the thyroid gland and an air pocket near the pyriform sinus. Pharyngoscopy revealed swelling of the left arytenoid region, with purulent retention. The left vocal cord was swollen but not paralyzed. Additionally, the laboratory data indicated thyrotoxicosis. Suspecting a PSF infection, parenteral treatment with cefotaxime and dexamethasone was initiated. On the following day, the hoarseness disappeared, and the fever resolved. Four weeks after onset, the thyroid hormone levels returned to the normal range, and a barium esophagogram revealed residual contrast in the left pyriform sinus, leading to a diagnosis of PSF.

**Conclusion:**

PSF presenting with hoarseness as the first symptom in patients should be considered.

## Background

Pyriform sinus fistulas (PSFs) are rare congenital anomalies caused by incomplete obliteration of the third or fourth brachial pouch [1]. PSFs often present with recurrent fever, neck pain, and swelling near the thyroid gland. They more commonly occur in neonates and young children and do not differ by sex [2, 3]. The left side of the neck is the predominantly affected site. Most patients present with a neck abscess and acute suppurative thyroiditis.

Dyspnea may appear secondary to lesion compression due to a neck mass [2, 4]. However, hoarseness has not been reported as the first symptom of a PSF. Herein, we report a case of an 11-year-old girl with a PSF presenting with hoarseness as the first symptom.

## Case presentation

**Fig. 1:**
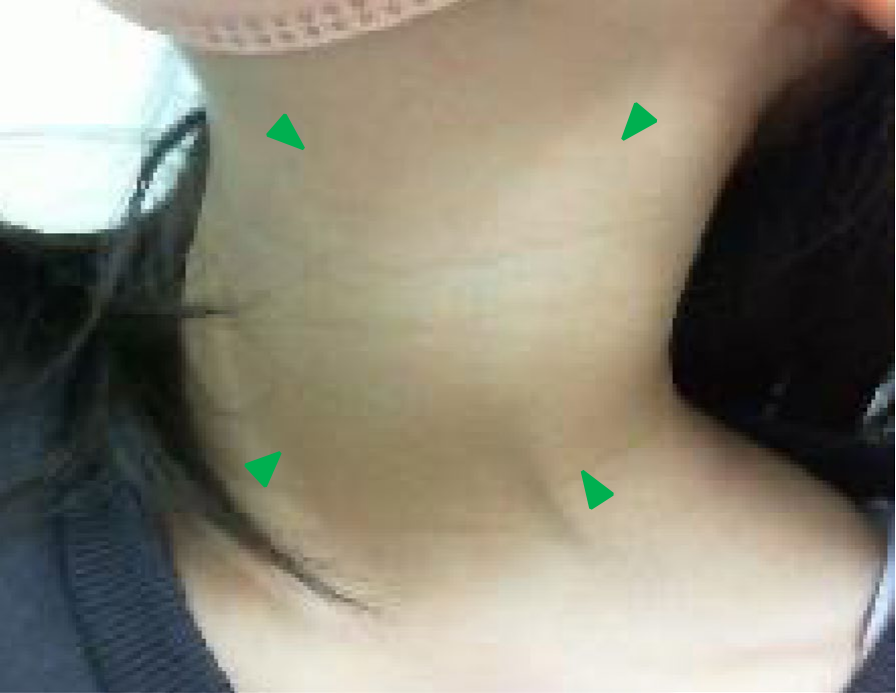
The left anterior neck on admission. An elastic-soft mass with mild tenderness on the left anterior neck

**Fig. 2:**
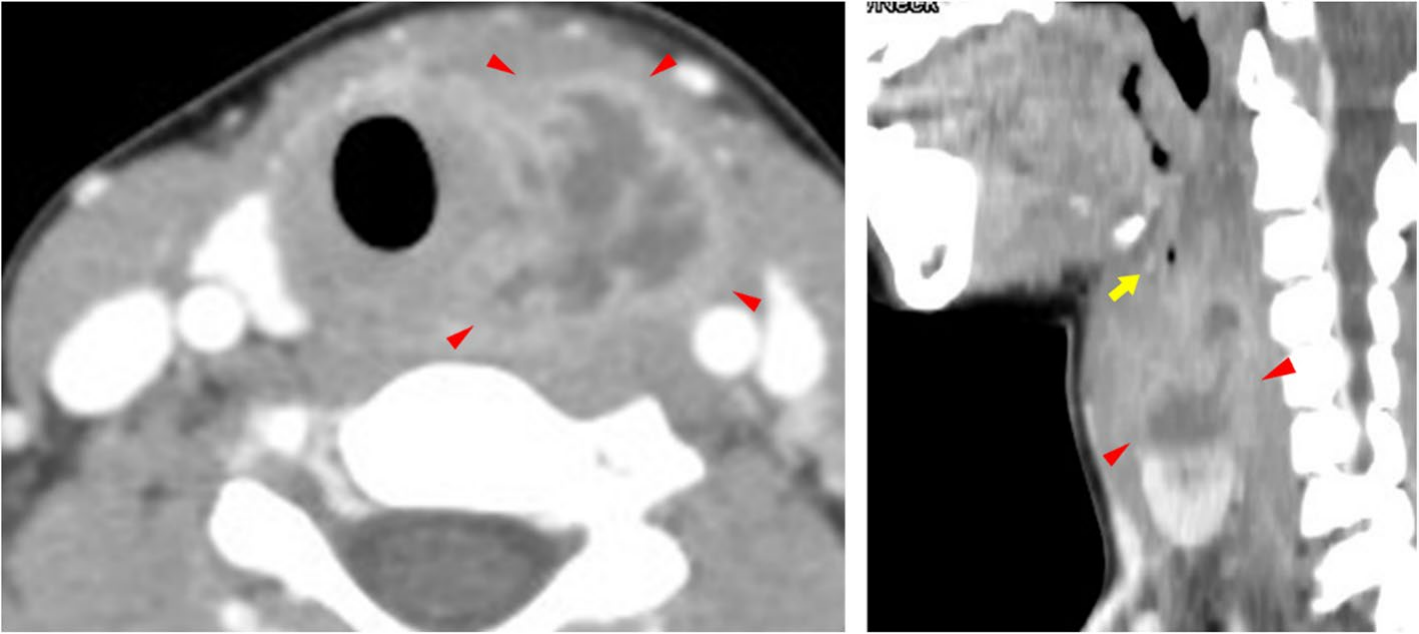
Contrast-enhanced computed tomography (CT) of the cervical region on admission. The contrast-enhanced CT scan of the cervical region revealed an abscess partially infiltrating the thyroid gland (arrowhead) and an air pocket (arrow)

**Fig. 3:**
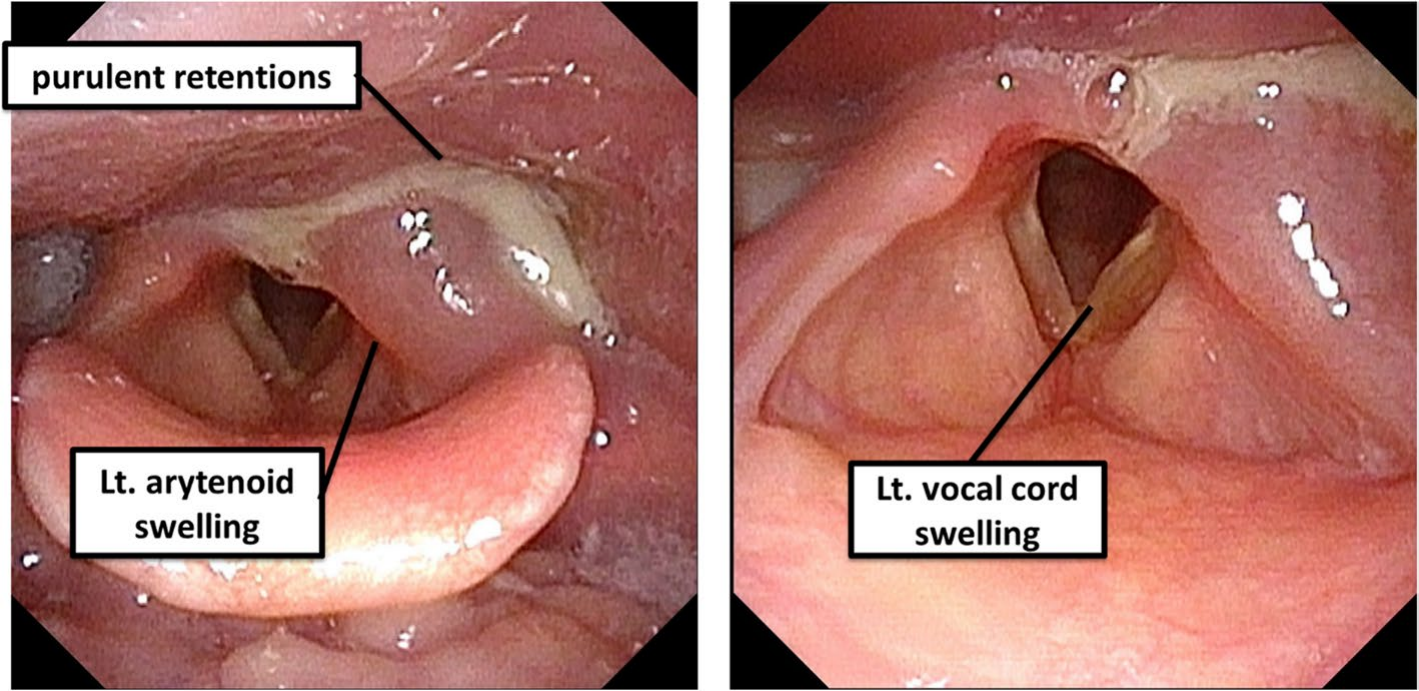
Pharyngoscopy examined on first day of admission. Pharyngoscopy revealed swelling of the left vocal cord and arytenoid region with purulent retentions as well as normal movement of the vocal cords (first day of admission)

**Fig. 4:**
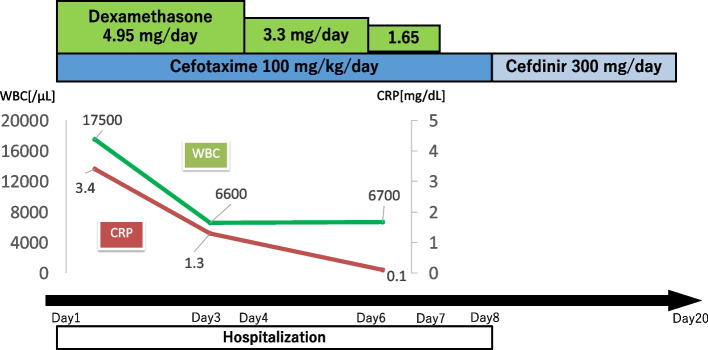
Treatment course

**Fig. 5:**
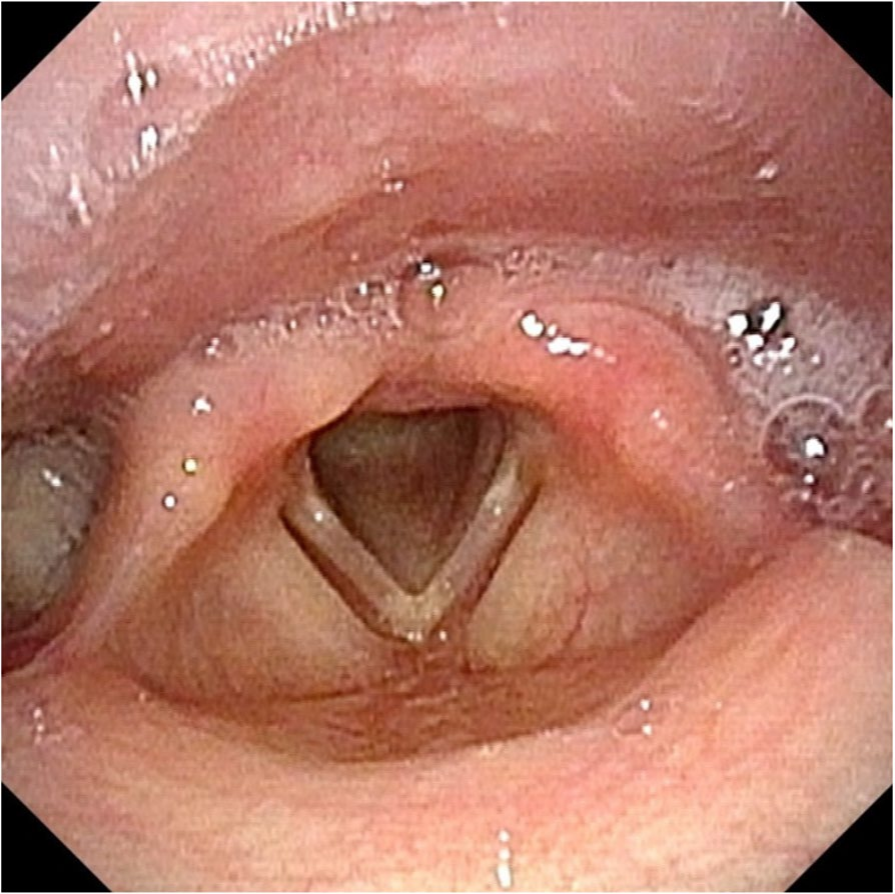
Pharyngoscopy examined on third day of admission. Pharyngoscopy revealed that the swelling disappeared (third day of admission)

An 11-year-old girl was referred to our hospital with the chief complaint of a swollen left-sided neck mass. Two days prior, hoarseness began in the morning, followed by fever and sore throat in the afternoon. On the next day, neck discomfort with tenderness occurred. On admission, she had a fever of 38.2 °C, hoarseness, and an elastic soft mass with mild tenderness on the left anterior neck (Fig. 1). Pharyngeal redness or swollen tonsils were not observed. Laboratory data indicated mild inflammation and thyrotoxicosis; increased white blood cell of 17,500/mm^3^ (neutrophils, 86.6% and lymphocytes, 8.6%) and C-reactive protein level of 3.4 mg/dL; increased free T4 of 1.98 ng/dL [reference 1.02~1.52 ng/dL], decreased free T3 and thyroid-stimulating hormone (TSH) level of 2.71 pg/mL [reference 2.78~4.90 pg/mL] and 0.009 μIU/mL [reference 0.62~3.36 μIU/mL] respectively; and an elevated thyroglobulin level of 308 ng/mL [reference 0~33.7 ng/mL]. Contrast-enhanced computed tomography (CT) of the cervical region revealed an abscess partially infiltrating the thyroid gland and an air pocket near the piriform sinus (Fig. 2). Pharyngoscopy revealed swelling of the arytenoid region, with purulent retention (Fig. 3). From the purulent discharge, Klebsiella oxytoca was isolated. The left vocal cord was swollen but not paralyzed. No evidence of airway narrowing was identified. Suspecting PSF infection, parenteral treatment with cefotaxime at 100 mg/kg/day and dexamethasone (DEX) at 0.16 mg/kg/day was initiated (Fig. 4). On the day after admission, the hoarseness disappeared, and the fever resolved. On the third day of admission, pharyngoscopy revealed that the swelling had disappeared (Fig. 5). DEX was tapered off within 5 days. On the seventh day of admission, a subsequent contrast-enhanced CT of the cervical region revealed a prominent reduction in the abscess. The patient was discharged on the eighth day of admission, and the antibiotic was switched to oral cefdinir 10 mg/kg/day.

**Fig. 6:**
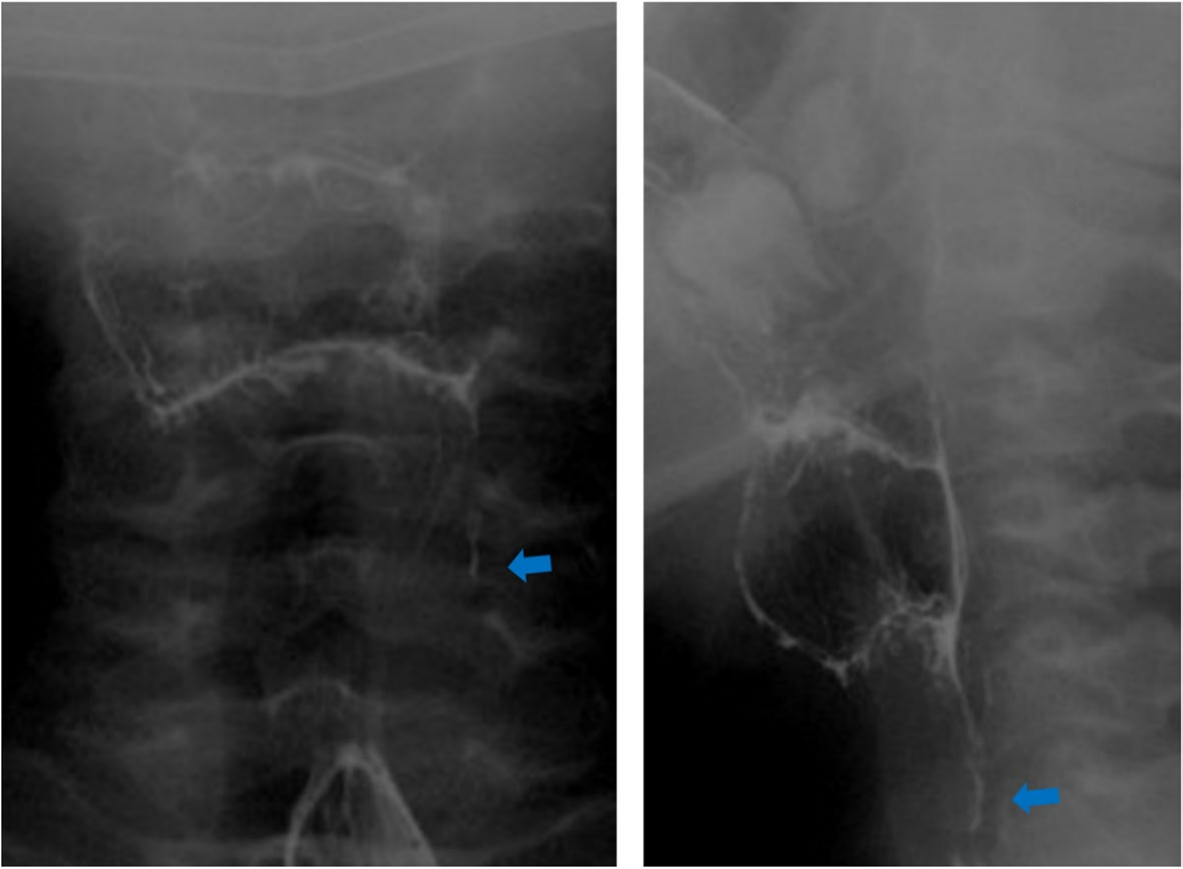
Barium esophagogram examined 3 weeks after discharge. A barium esophagogram revealed a residual contrast in the left pyriform sinus (arrow)

Three weeks after discharge, a barium esophagogram revealed residual contrast in the left pyriform sinus, and PSF was diagnosed (Fig. 6). However, abscess was not detected by ultrasonography. At that time, thyroid function returned to the normal range (FT3, 4.19 pg/ml; FT4, 1.19 ng/dl; TSH level, 2.63 μIU/mL; and thyroglobulin level, 16.4 ng/mL). Chemocauterization was proposed to the patient’s family because this was the first episode of a neck abscess. However, the family did not opt for this method, and the patient is currently under observation without recurrence for a year.

## Discussion and conclusions

The initial symptoms of PSF include neck mass with or without abscess, acute thyroiditis, and thyroid lesion [1–3]. In some cases, particularly in neonates, a neck mass compressing the surrounding structure, resulting in dyspnea, has been reported [2, 4]. However, hoarseness, as the first symptom of PSF, has not yet been reported. This study presents a case of PSF with hoarseness as the first symptom preceding fever and neck swelling. Flexible laryngoscopy, performed to evaluate hoarseness, revealed swelling of the arytenoid region with purulent retention. The left vocal cord was swollen but not paralyzed. Hoarseness disappeared with DEX on the day after admission, and swelling disappeared on the third day. Thus, hoarseness may be derived from the swelling of the left vocal cord due to the PSF infection. As vocal cord movement was normal, the hoarseness was not caused by a recurrent laryngeal nerve palsy. Dyspnea has been reported to be secondary to lesion compression in some patients with PSF. However, in our case, no evidence of airway obstruction was identified, although hoarseness, fever, and anterior neck swelling were noted. Hence, the larynx and airways in patients with hoarseness need to be evaluated.

Acute suppurative thyroiditis is a rare clinical condition in childhood because the thyroid gland is remarkably resistant to infections owing to its high iodine content, rich blood and lymphocyte supply, and protective fibrous capsule [5]. Infection with PSF is often reported to cause acute suppurative thyroiditis [1, 6]. In our case, a low TSH level and high FT4 and TG levels suggested that the abscess had partially destroyed the thyroid gland. Considering the suppressed TSH level, high FT3 level may have occurred before admission, although the FT3 level on admission was low. The presence of complicated low T3 syndrome due to infection and decreased intake was indicated. A previous study has reported that low T3 syndrome was associated with acute suppurative thyroiditis because of PSF infection in an adult case [7]. The thyroid hormone levels returned to the normal range within 3 weeks.

Diagnosing PSF can be difficult owing to its rarity, and PSF should be considered when examining neck abscesses or acute suppurative thyroiditis. In the acute phase of infection, identifying the fistula tract is difficult because of swelling of the mucosa and surrounding tissues [6]. In our case, acute-phase contrast-enhanced CT revealed an abscess partially infiltrating the thyroid gland and an air pocket near the pyriform sinus. The air pocket resembled a ductal structure from the pyriform sinus to the thyroid, which was elevated by the abscess, thus suggesting the presence of a PSF. After treatment of the acute inflammation, we confirmed the result with barium esophagogram, confirming the diagnosis of a PSF. As the diagnosis had already been made, we didn’t perform direct laryngoscopy under general anesthesia.

Antibiotics and percutaneous drainage are commonly used to treat PSFs in the acute phase of infection [2]. Surgical intervention for drainage of abscess and closure of branchial arch sinus was proposed to the patient’s family at admission, but the family didn’t choose the methods, and the patient was treated with antibiotics. In addition, corticosteroids were administered to improve the laryngeal swelling. Surgical fistula removal is necessary for treating PSFs [2]. Obliteration of the inner orifice by chemocauterization, laser coagulation, and biocauterization is also effective in the management of PSFs [8]. However, a PSF may not be entirely closed through these methods. Therefore, complete fistula resection is a reasonable treatment method for recurrent cases. In the present case, although we recommended chemocauterization because it was the first episode, the patient’s family did not select this method, and the patient is currently under observation without recurrence for a year.

This study describes a rare case of PSF with acute suppurative thyroiditis with hoarseness arising as the first symptom. Therefore, the possibility of PSF in patients presenting with hoarseness as their first symptom should be considered.

## Data Availability

The data supporting the findings of this study are available from the corresponding author upon reasonable request.

## Abbreviations

CT: Computed tomography
DEX: Dexamethasone
PSF: Pyriform sinus fistula
TSH: Thyroid-stimulating hormone
